# 外泌体miR-10b通过调控巨噬细胞M2极化促进肺腺癌A549细胞的侵袭和上皮间质转化

**DOI:** 10.3779/j.issn.1009-3419.2022.101.50

**Published:** 2022-12-20

**Authors:** 燕 袁, 利敏 郭, 珊 郭

**Affiliations:** 453000 新乡，新乡市中心医院 Xinxiang Central Hospital, Xinxiang 453000, China

**Keywords:** A549, M2型巨噬细胞极化, 外泌体, miR-10b, 侵袭, 上皮间质转化, A549, M2 macrophage polarization, Exosome, miR-10b, Invasion, Epithelial mesenchymal transition

## Abstract

**背景与目的:**

肺癌转移是引起死亡的主要原因。先天免疫细胞特别是巨噬细胞在肿瘤转移中扮演非常重要的角色。外泌体在肿瘤细胞与巨噬细胞间的通讯非常重要，因此本研究探讨肺腺癌A549细胞外泌体中的miR-10b对其细胞生长侵袭及上皮间质转化（epithelial mesenchymal transition, EMT）的影响。

**方法:**

从A549细胞中分离外泌体，通过透射电镜及蛋白质印迹法对外泌体进行鉴定。采用CCK-8试剂盒和流式细胞术检测A549细胞的增殖和凋亡。Transwell法检测细胞侵袭以及通过定量逆转录聚合酶链式反应（quantitative reverse transcription polymerase chain reaction, RT-qPCR）和Western blot分别检测A549细胞中mRNA和蛋白的表达。

**结果:**

miR-10b在非小细胞肺癌（A549、NCI-H1650和NCI-H1299）中表达上调，miR-10b inhibitor可抑制非小细胞肺癌的增殖。同时，外泌体miR-10b通过促进巨噬细胞M2极化，进而促进A549细胞的侵袭和EMT。

**结论:**

外泌体miR-10b通过M2巨噬细胞极化促进A549细胞的侵袭和EMT。

## Abstract (en)

**Background and objective:**

Metastasis is the main cause of death in patients with lung cancer. Macrophages are innate immune cells that play important roles in cancer metastasis. Exosomes could play an important role of communication between tumor cells and macrophages. This study investigated the effect of miR-10b on cell growth invasion and epithelial mesenchymal transition (EMT) in lung adenocarcinoma A549 cell exosomes.

**Methods:**

Exosomes were isolated from A549 cells and identified by transmission electron microscopy (TEM) and Western blot. CCK-8 assay and flow cytometry were used to detect cell proliferation and apoptosis. Cell migration and invasion were detected by Transwell assay. The expression of mRNA and protein were assessed by quantitative reverse transcription polymerase chain reaction (RT-qPCR) and Western blot, respectively.

**Results:**

The expression of miR-10b was up-regulated in non-small cell lung cancer, and miR-10b inhibitor could inhibit the proliferation of A549 cell. Meanwhile, the tumor cell-derived exosome miR-10b promoted the invasion of A549 cell and EMT by promoting the M2 polarization of macrophages.

**Conclusion:**

Tumor cell-derived exosome miR-10b promotes A549 cell invasion and EMT through M2 macrophage polarization.

肺癌是全球最常见的恶性肿瘤，死亡率居恶性肿瘤之首，其中约85%为非小细胞肺癌（non-small cell lung cancer, NSCLC）。NSCLC的治疗备受关注，但效果仍不理想^[1, 2]^。因此，有必要寻找新的治疗NSCLC的方法。先前的研究^[3]^表明，肿瘤微环境（tumor microenvironment, TME）中的肿瘤细胞和间质细胞之间的联系在调节癌症的发展中起着关键作用。越来越多的证据^[4, 5]^表明，外泌体通过将信号肽、非编码RNA或DNA转移到邻近的细胞或组织，在重塑TME和肿瘤转移中发挥关键作用。存在于外泌体中的microRNAs（miRNAs）可以被邻近或远处的细胞吸收，它们随后调节受体细胞。miRNAs是内源性的具有调控功能的非编码RNA，从而调控各种癌症的进展^[6]^。外泌体miRNA的失调可以影响癌细胞和TME之间的串扰。巨噬细胞是肿瘤内及周围最丰富的浸润性免疫相关的基质细胞，表现出不同的表型和功能。它们可以被不同刺激极化为经典激活或炎症（M1）和交替激活或抗炎（M2）巨噬细胞^[7, 8]^。肿瘤相关巨噬细胞（tumor-associated macrophages, TAMs）被认为是M2表型存在于中，通过与癌细胞相互作用影响多种癌症的转移。癌细胞与M2巨噬细胞之间的串扰已被广泛研究^[9]^。然而，NSCLC治疗与TAMs之间的详细联系尚不清楚。先前多项研究发现miR-10b在NSCLC组织中的表达高于癌旁正常组织中的表达^[10, 11]^，且已有研究指出肝细胞癌外泌体miR-10b促进癌细胞增殖和转移^[12]^，但其在肺癌发生及进展中的作用和机制尚不清楚。因此，本研究通过差速离心法提取肺腺癌A549细胞的外泌体并探讨外泌体中的miR-10b对巨噬细胞极化状态的影响，并探究发生M2型极化巨噬细胞对A549细胞侵袭和上皮间质转化的作用，阐明外泌体miRNA可通过改变TME中巨噬细胞的极化状态而影响肿瘤的发生发展，可作为潜在的肿瘤生物标志物。

## 1 材料与方法

### 1.1 材料

NSCLC细胞系（A549、NCI-H1650和NCI-H1299）、BEAS-2B和THP-1细胞购自中国科学院上海细胞库，DMEM高糖培养基、青/链霉素双抗、0.25%胰蛋白酶、1×PBS缓冲液（Gibco），Lipofectamine 2000购自Invitrogen公司；miR-10b inhibitor/mimic和Cy3标记的miR-10b mimics购自上海吉玛基因公司，CCK-8检测试剂盒购自上海东仁化学科技有限公司，ECL-plus化学发光剂购自GE公司。

### 1.2 细胞培养与转染

细胞培养在含有10%胎牛血清和1%的链霉素和青霉素的DMEM培养基中，置于培养箱中（37 ℃、5%CO_2_）培养。细胞转染Cy3标记的miR-10b，miR-10b inhibitor/mimic或inhibitor/mimic control，使用Lipofectamine 2000（Thermo Fisher）按说明书步骤转染48 h，用于后续实验。

### 1.3 外泌体提取与鉴定

收集NSCLC细胞上清（300×*g*离心15 min，2, 000×*g*离心15 min，10, 000×*g*离心30 min）。随后，过滤细胞上清液并收集，通过超离心（120, 000×*g*离心70 min）分离外泌体。外泌体的结构由透射电镜观察，外泌体标记物（CD9、CD63、TSG101）由Western blot检测。

### 1.4 巨噬细胞的诱导极化

用佛波酯PMA（MCE）处理THP-1细胞，使其分化为巨噬细胞，再用M2诱导剂[白细胞介素4（interleukin-4, IL-4）20 ng/mL]处理24 h后观察细胞形态，得到极化的M2型巨噬细胞。

### 1.5 CCK8检测细胞存活率

收集各组细胞，将细胞种于96孔板中，密度为5×10^3^个/孔，按不同处理分为三组：Control组、Inhibitor control组、miR-10b inhibitor组，经过24 h培养后加入10 µL的CCK-8溶液，37 ℃孵育4 h，用酶标仪测定吸光度（450 nm）。

### 1.6 酶联免疫吸附法（enzyme linked immunosorbent assay, ELISA）检测细胞因子

细胞因子IL-6、肿瘤坏死因子α（tumor necrosis factor α, TNF-α）、IL-10和转化生长因子β（transforming growth factor β, TGF-β）的蛋白水平通过相对应的ELISA试剂盒并依据说明书进行检测，试剂盒购于南京森贝伽生物科技有限公司。

### 1.7 流式细胞仪检测细胞凋亡

收集各种细胞以1, 000 rpm离心3 min至沉淀细胞。随后，用5 μL Annexin V-FITC和PI避光染色15 min，流式细胞仪检测细胞凋亡情况。数据通过FlowJo进行量化。

### 1.8 Transwell小室分析

在涂有基质胶的Transwell小室中进行细胞侵袭实验。将细胞接种在6孔板中进行转染。转染48 h后，将细胞接种到涂有基质胶的侵入室的上隔室。孵育16 h后，将侵入插入物底部的细胞染色并在显微镜下计数。

### 1.9 免疫荧光

NSCLC细胞在甲醇中固定20 min，然后用anti-CD63（1:200; Abcam）或anti-PKH26（1:2, 000; Abcam）。之后，细胞与二抗孵育（1:5, 000；羊抗兔IgG，Abcam）。最后，用显微镜观察（Olympus）。

### 1.10 定量逆转录聚合酶链式反应（quantitative reverse transcription polymerase chain reaction, RT-qPCR）

采用TRIzol试剂（Takara）从细胞系中分离总RNA。使用PrimeScript逆转录试剂盒（Thermo Fisher）将总RNA逆转录成cDNA。随后，使用SytoxGreen试剂盒（Thermo Fisher）进行RT-qPCR。

### 1.11 蛋白免疫印迹法（Western blot）检测LDGs中CitH3及MPO的表达水平

采用RIPA裂解缓冲液（Beyotime）从细胞中分离总蛋白。采用BCA试剂盒（Beyotime）定量总蛋白。使用SDS-PAGE（10%）分离蛋白，然后将蛋白转移到PVDF膜上（Thermo Fisher）。用5%脱脂牛奶封闭1 h后，细胞膜与一抗孵育过夜：anti-CD63（Abcam; 1:1, 000）、anti-TSG101（Abcam; 1:1, 000）、anti-CD81（Abcam; 1:1, 000）、anti-E-cadherin（Abcam; 1:1, 000）、anti-N-cadherin（Abcam; 1:1, 000）和抗anti-β-actin（Abcam; 1:5, 000）。之后用相应的二抗孵育，最终使用ECL试剂盒来观察蛋白条带，Image J软件进行蛋白条带的灰度分析。

### 1.12 统计学处理

采用GraphPad Prism软件进行统计分析。所有数据采用均数±标准差（Mean±SD）。显著性采用SPSS 18.0软件进行分析。组间比较用单因素方差分析，多重比较采用*t*检验。*P* < 0.05为差异有统计学意义。

## 2 结果

### 2.1 miR-10b在NSCLC系中的表达水平

为了研究miR-10b在NSCLC细胞中的作用，通过RT-qPCR检测miR-10b的表达。如图 1A所示，与BEAS-2B细胞相比，miR-10b在A549、NCI-H1299和NCI-H1650细胞中的表达明显上调，其中A549细胞中表达水平最高。因此我们选择其作为后续研究的对象，同时，与对照组相比miR-10b inhibitor转染的A549细胞中miR-10b水平显著下调（图 1B）。此外，CCK8结果显示抑制miR-10b的表达显著降低A549细胞的存活率（图 1C）。

**图 1:**
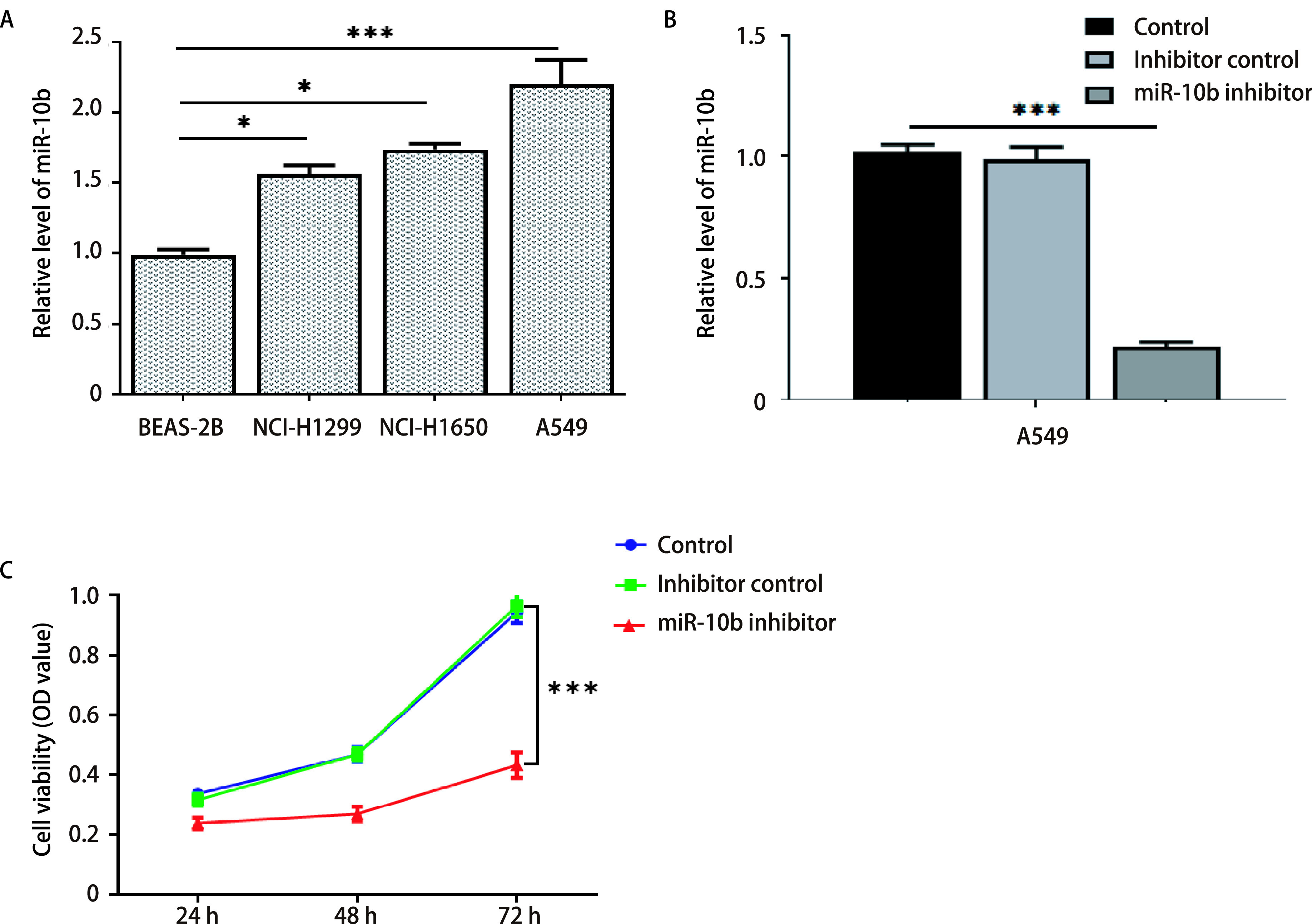
MiR-10b在NSCLC中的表达。A：RT-qPCR检测BEAS-2B、NCI-H1650、NCI-H1299和A549细胞中miR-10b的表达水平；B：A549细胞转染inhibitor control或miR-10b inhibitor。然后用RT-qPCR检测miR-10b在A549细胞中的表达；C：采用CCK-8法检测A549细胞的生存能力。^*^P < 0.05；^***^P < 0.001。 Expression level of miR-10b in NSCLC. A: The level of miR-10b in BEAS-2B, NCI-H1650, NCI-H1299 or A549 cells was detected by RT-qPCR; B: A549 cells were transfected with inhibitor control or miR-10b inhibitor. Then, the expression of miR-10b in A549 cells was tested by RT-qPCR. C: The viability of A549 cells was tested by CCK-8 assay. ^*^P < 0.05; ^***^P < 0.001. RT-qPCR: quantitative reverse transcription polymerase chain reaction; NSCLC: non-small cell lung cancer.

### 2.2 miR-10b可以通过外泌体从A549细胞转移到巨噬细胞

据报道，肿瘤来源的外泌体在肿瘤发生中发挥重要作用。因此，我们试图从A549细胞中分离外泌体，然后用透射电子显微镜（transmission electron microscope, TEM）观察，结果如图 2A所示，所提取的外泌体具有双层膜结构，呈典型的“杯口状”结构。此外，与A549细胞相比，A549-exo的外泌体蛋白（TSG101、CD63和CD81）的表达显著升高（图 2B）。同时RT-qPCR检测结果表明与A549细胞相比miR-10b的表达在A549-exo中表达显著升高（图 2C）。如图 2D所示，红色荧光为Cy3标记的miR-10b，蓝色荧光为THP-1来源的巨噬细胞的细胞核，在巨噬细胞胞浆中可见红色荧光，提示：miR-10b可由外泌体作为传递介质，被巨噬细胞摄取。此外，利用Transwell小室建立共培养模型（图 2E），上室为供体细胞A549，下室为受体细胞M0型巨噬细胞，供体细胞所分泌的外泌体通过小室膜进入受体细胞，而细胞不会通过该孔径。如图 2F结果显示，共培养24 h后分别收集供体细胞与受体细胞，通过qRT-PCR结果显示供体细胞过表达miR-10b后，可通过外泌体进入受体细胞中，促进受体细胞中miR-10b的表达水平（图 2F）。

**图 2:**
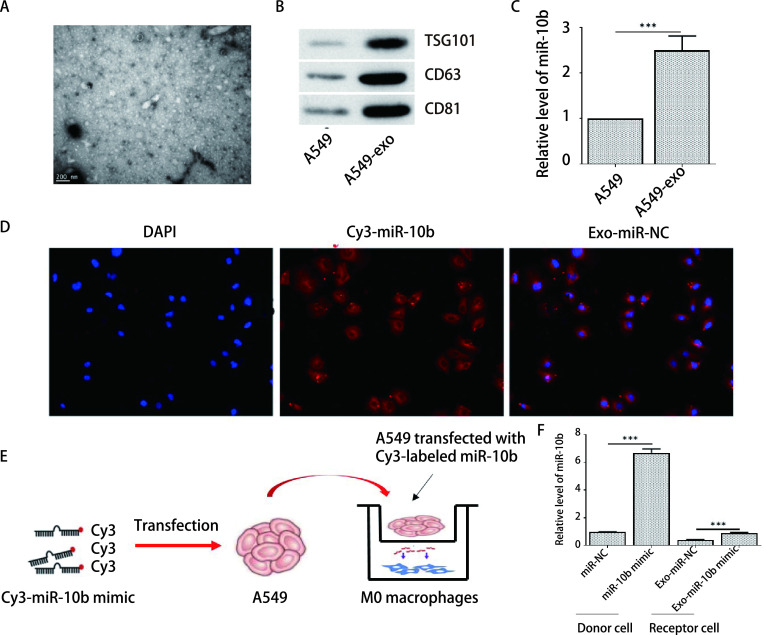
MiR-10b可以通过外泌体从A549细胞转移到巨噬细胞。A：TEM观察到的外泌体；B：Western blot检测A549细胞中TSG101、CD63和CD81的蛋白表达；C：RT-qPCR检测miR-10b的表达；D：免疫荧光染色观察外泌体中Cy3-miR-10b被巨噬细胞的摄取（DAPI：巨噬细胞核；Cy3：外泌体中miR-10b）；E：共培养模型示意图；F：RT-qPCR检测miR-10b的表达。^***^P < 0.001。 MiR-10b can be transferred from A549 cells to macrophages via exosomes. A: The separation efficiency of exosomes was examined by TEM; B: The expressions of TSG101, CD63 and CD81 inA549 cells were detected by Western blot; C: The expression of miR-10b was detected by RT-qPCR. D: Immunofluorescence staining was used to observe the uptake of Cy3-miR-10b by macrophages in exosomes (DAPI: macrophage nucleus; CY3: miR-10b in exosomes); E: Schematic diagram of co-culture model; F: The expression of miR-10b was detected by RT-qPCR. ^***^P < 0.001. Cy3: Sulfo-Cyanine 3; Exo: exosome; TEM: transmission electron microscope.

### 2.3 外泌体miR-10b促进巨噬细胞M2极化

为研究外泌体miR-10b对THP-1源巨噬细胞的极化影响，我们设立4个实验组：A549细胞转染miR-NC与巨噬细胞共培养组（Exo-miR-NC组）、A549细胞转染miR-10b mimic与巨噬细胞共培养组（Exo-miR-10b组）、M2型巨噬细胞组（M2 macrophage组）、M2型巨噬细胞+miR-10b inhibitor组（M2+miR-10b inhibitor组），从正反两个方面验证miR-10b对M2型巨噬细胞的极化作用。如图 3A所示，Exo-miR-10b组M2型巨噬细胞标志物（Arginase-1、IL-10、CD206、CD209和TGF-β）的表达水平较Exo-miR-NC组显著升高。而对M2型巨噬细胞降低miR-10b表达后，抑制了M2型巨噬细胞标志物的表达水平，从反向验证了miR-10b低表达对M2型巨噬细胞的抑制作用。同时ELISA检测细胞因子IL-6、TNF-α、IL-10和TGF-β的蛋白水平，结果与mRNA表达水平一致，这些结果表明miR-10b可以促进M2巨噬细胞的极化（图 3B）。

**图 3:**
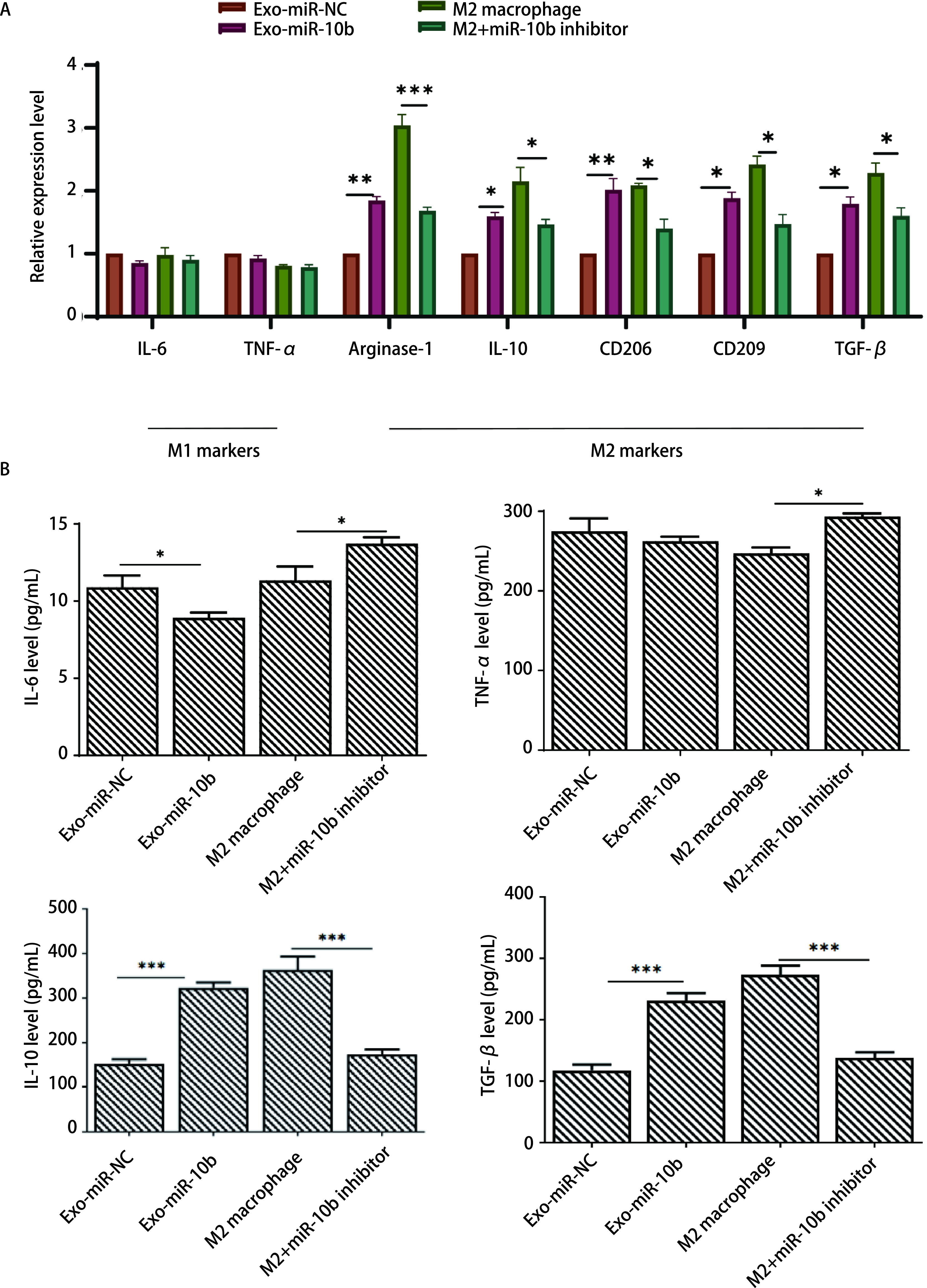
外泌体miR-10b促进巨噬细胞M2极化。A：RT-qPCR检测巨噬细胞IL-6、TNF-*α*、Arginase-1、IL-10、CD206、CD209、TGF-*β*水平；B：采用ELISA检测巨噬细胞上清液中IL-6、TNF-*α*、TGF-*β*和IL-10水平。^*^P < 0.05；^**^P < 0.01；^***^P < 0.001。 Exosomal miR-10b promoted M2 polarization in macrophages. A: The levels of IL-6, TNF-*α*, Arginase-1, IL-10, CD206, CD209 and TGF-*β* in macrophages were tested by RT-qPCR. B: The levels of IL-6, TNF-*α*, TGF-*β* and IL-10 in supernatants of macrophages were detected by ELISA. ^*^P < 0.05; ^**^P < 0.01; ^***^P < 0.001. IL: interleukin; TNF: tumor necrosis factor; TGF: transforming growth factor; NC: negative control; ELISA: enzyme linked immunosorbent assay.

### 2.4 外泌体miR-10b通过促进M2巨噬细胞的极化调控A549细胞侵袭和上皮间质转化（epithelial mesenchymal transition, EMT）过程

为了研究外泌体miR-10b是否通过促进M2巨噬细胞极化来调控A549细胞的侵袭和EMT，首先进行Transwell实验。如图 4A所示，Exo-miR-10b组细胞侵袭能力增强。同时，对于M2型巨噬细胞，抑制miR-10b表达水平细胞侵袭能力显著降低。同时，与Exo-miR-NC组相比，miR-10b表达上调的外泌体能显著降低E-cadherin的表达和升高N-cadherin的表达水平。对于M2型巨噬细胞，抑制miR-10b表达水平，细胞EMT转化水平受到抑制（图 4B）。综上所述，外泌体miR-10b可以通过促进M2巨噬细胞的极化来调控A549细胞的侵袭和EMT过程。

**图 4:**
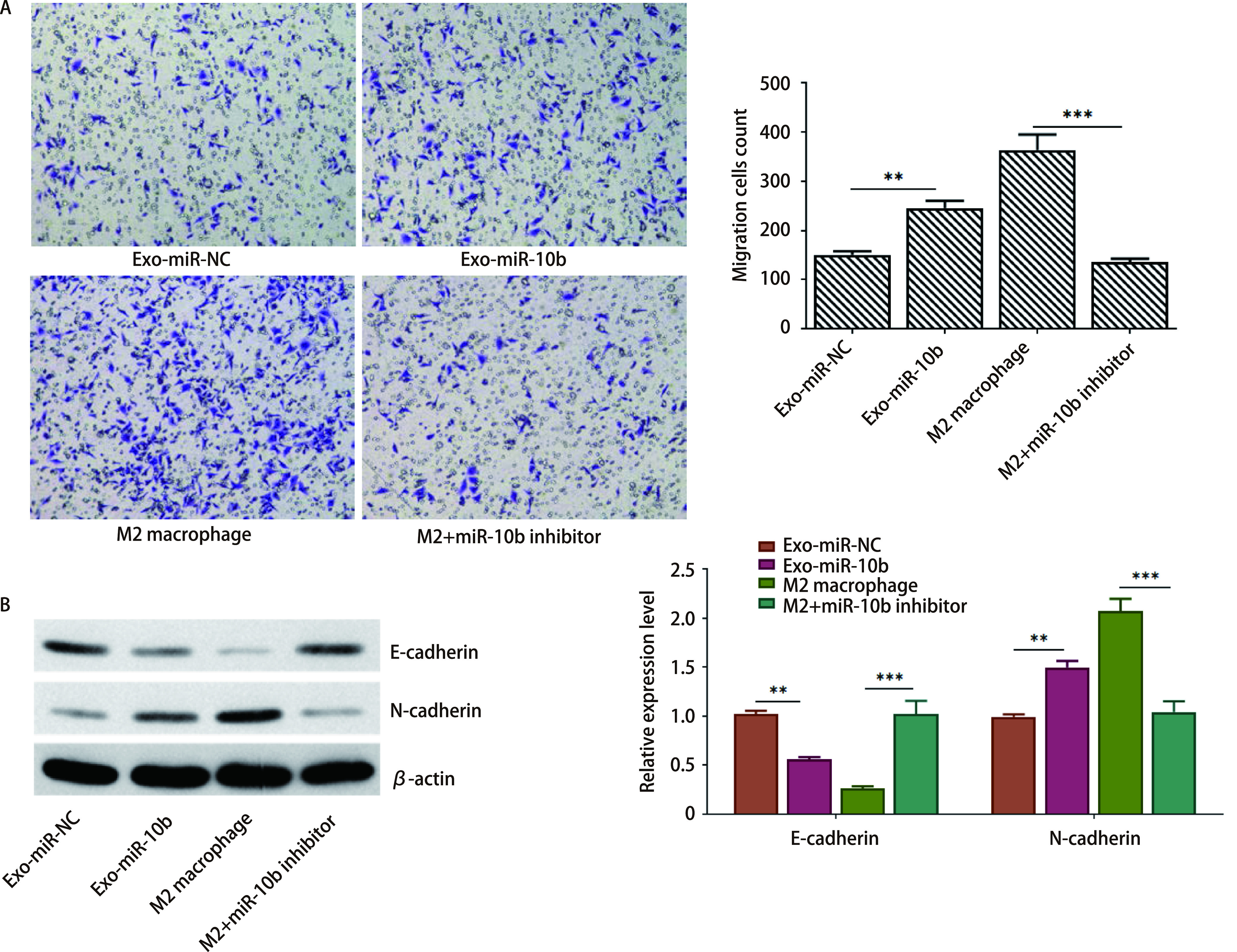
外泌体miR-10b通过促进M2巨噬细胞的极化调控A549细胞的侵袭和EMT过程。A：A549细胞的迁移采用Transwell检测；B：Western blot检测A549细胞中E-cadherin和N-cadherin蛋白水平。^**^P < 0.01；^***^P < 0.001。 Exosomal miR-10b significantly promoted the migration and EMT process of A549 cells via regulating the polarization of M2 macrophages. A: The migration of A549 cells was tested by transwell assay; B: The protein levels of E-cadherin and N-cadherin in A549 cells were detected by Western blot. ^**^P < 0.01; ^***^P < 0.001. EMT: epithelial mesenchymal transition.

## 3 讨论

肺癌细胞的侵袭和转移是其恶化的重要原因，也是影响患者治疗效果及导致死亡的主要原因。以往的报道表明TME中的巨噬细胞可通过促进血管生成、促进癌细胞迁移和侵袭、抑制机体的抗肿瘤免疫反应，加重恶性肿瘤的进展^[13, 14]^。研究^[15]^发现，肺癌细胞与巨噬细胞共培养更容易转移到肝、肺和骨组织。然而，巨噬细胞主要极化为M1或M2类表型，且其功能在TME中各不相同，M1巨噬细胞通常发挥肿瘤抑制功能，而M2巨噬细胞则发挥肿瘤促进功能。重要的是，Guo等^[16]^发现与LUAD细胞共培养可导致THP1巨噬细胞向恶性方向发展，促进M2极化。这些报道表明，在肺癌微环境中，诱导M2极化可促进肿瘤转移，因此我们研究巨噬细胞M2极化对肺癌细胞A549生物学功能的影响。外泌体由多种类型的细胞分泌（尤其是肿瘤细胞），据报道它们与癌症进展密切相关^[17]^。此前的研究^[18, 19]^显示，外泌体可以构建一种微环境，通过癌细胞和周围基质细胞之间的关联诱导癌症进展。在本研究中，肺腺癌细胞A549来源的外泌体miR-10b促进巨噬细胞M2极化，从而导致A549细胞的侵袭和EMT能力增强。因此，TME中肿瘤细胞分泌的外泌体可改变TME，影响肿瘤的发生发展过程。

有报道^[20]^称，miRNAs的失调往往会导致癌症的发生。miRNA是一类小分子非编码RNA，在转录后水平起着重要的基因调控作用。其中，通过外泌体传递的miRNAs介导了肿瘤细胞和巨噬细胞之间的交流^[21]^。几种外泌体miRNAs与M2样表型相关。胰腺癌中，低氧诱导的外泌体miR-301促进M2巨噬细胞极化^[22]^。此外，巨噬细胞的浸润受miR-28-5p和IL-34表达的影响，促进肝细胞癌的转移^[23]^。我们目前的研究发现miR-10b在肺腺癌A549细胞中显著升高，促进细胞的生长。miR-10b也可以通过直接靶向肺癌细胞中的p53调节顺铂的耐药性^[24]^。与此同时，外泌体中过表达miR-10b能促进M2巨噬细胞的极化，从而促进A549细胞的迁移侵袭和EMT过程。MiR-10b在肿瘤发生和转移中具有重要的生物学功能^[25]^。有研究^[26]^发现胞外囊泡中miR-10b可能是优于血浆miR-10b的肺腺癌诊断的潜在标志物。Yuan等^[27]^发现M1巨噬细胞抑制肿瘤生长和血管生成，增强化疗敏感性，M2巨噬细胞促进癌细胞侵袭。这些数据表明A549细胞来源的外泌体miR-10b在TME中发挥关键作用，对肿瘤转移至关重要。

综上所述，本研究初步证实A549细胞能够通过分泌高表达miR-10b的外泌体调控M2巨噬细胞的极化促进A549细胞的侵袭和EMT过程。
